# Protective Effect of *Fragaria ananassa* Crude Extract on Cadmium-Induced Lipid Peroxidation, Antioxidant Enzymes Suppression, and Apoptosis in Rat Testes

**DOI:** 10.3390/ijms18050957

**Published:** 2017-05-05

**Authors:** Mohammed I. Y. Elmallah, Manal F. Elkhadragy, Ebtesam M. Al-Olayan, Ahmed E. Abdel Moneim

**Affiliations:** 1 Chemistry Department, Faculty of Science, Helwan University, Cairo 11795, Egypt; Mohamed_almallah@science.helwan.edu.eg; 2 Marine Natural Product Unit (MNPRU), Faculty of Science, Helwan University, Cairo 11795, Egypt; 3 Department of Zoology, College of Science, King Saud University, Riyadh 11451, Saudi Arabia; manalelkhadragy@yahoo.com (M.F.E.); eolayan@hotmail.com (E.M.A.-O.); 4 Department of Zoology and Entomology, Faculty of Science, Helwan University, Cairo 11795, Egypt; 5 Chair Vaccines Research of Infectious Diseases, Faculty of Science, King Saud University, Riyadh 11472, Saudi Arabia

**Keywords:** *Fragaria ananassa*, oxidative stress, cadmium intoxication, testes, apoptosis

## Abstract

Cadmium is a deleterious environmental pollutant that threats both animals and human health. Oxidative stress and elevated levels of reactive oxygen species (ROS) have recently been reported to be the main cause of cellular damage as a result of cadmium exposure. We investigate, here, the protective effect of strawberry crude extracts on cadmium-induced oxidative damage of testes in rats. Four groups (*n* = 8) of 32 adult male Wistar rats weighing 160–180 g were used. The control group received 0.9% saline solution all over the experimental period (5 days). Group 2 was intraperitoneally injected with 6.5 mg/kg CdCl_2_. Group 3 was provided only with an oral administration of strawberry methanolic extract (SME) at a dose of 250 mg/kg. Group 4 was treated with SME before cadmium injection with the same mentioned doses. It was shown that cadmium exposure results in a significant decrease in both relative testicular weight and serum testosterone level. Analyzing the oxidative damaging effect of cadmium on the testicular tissue revealed the induction of oxidative stress markers represented in the elevated level of lipid peroxidation (LPO), nitric oxide (NO), and a decrease in the reduced glutathione (GSH) content. Considering cadmium toxicity, the level of the antioxidant enzyme activities including catalase (CAT), superoxide dismutase (SOD2), glutathione peroxidase (GPx1), and glutathione reductase (GR) were markedly decreased. Moreover, gene expression analysis indicated significant upregulation of the pro-apoptotic proteins, bcl-2-associated-X-protein (*BAX*), and tumor necrosis factor-α (*TNFA*) in response to cadmium intoxication, while significant downregulation of the anti-apoptotic, B-cell lymphoma 2 (*BCL2*) gene was detected. Immunohistochemistry of the testicular tissue possessed positive immunostaining for the increased level of TNF-α, but decreased number of proliferating cell nuclear antigen (PCNA) stained cells. Administration of SME debilitated the deleterious effect of cadmium via reduction of both LPO and NO levels followed by a significant enhancement in the gene expression level of *CAT*, *SOD2*, *GPX1*, *GR*, nuclear factor-erythroid 2-related factor 2 (*NFE2L2*), heme oxygenase-1 (*HMOX1*), Bcl-2, and PCNA. In addition, the SME treated group revealed a significant increase in the level of testosterone and GSH accompanied by a marked decrease in the gene expression level of Bax and TNF-α. In terms of the summarized results, the SME of *Fragaria ananassa* has a protective effect against cadmium-induced oxidative damage of testes.

## 1. Introduction

Heavy metals’ toxicity is considered to be one of the major threats to healthy life [1]. The degree of toxicity is mainly assigned to solubility and absorption status. Cadmium (Cd) is a naturally-distributed heavy metal, which can be found in water, air, and food as well as in cigarette smoke [2]. In addition, Cd has been reported to be a pollutant product of several industries, including the manufacture of batteries, dyes, paint pigments, plastics, fertilizers and some agricultural sources [3,4]. It binds to biological macromolecules like proteins; and some other compounds, such as metallothionein and sulfhydryl-containing molecules, are responsible for the protection of repair systems in living cells against free-radical-induced cell damage [2]. Several studies have revealed that Cd toxicity stimulates the production of reactive oxygen species (ROS) and the induction of oxidative stress in different organs [5]. These radicals are able to oxidize biological macromolecules, particularly proteins and DNA results in structural perturbations and subsequent metabolic disorders [6]. Moreover, cadmium exposure stimulates lipid peroxidation-induced tissue damage [7]. Subcutaneous administration of cadmium chloride in rabbits was found to affect specific organs, most frequently testes, kidneys, spleen, bone, and liver [4]. It has been reported that cadmium salts like cadmium chloride cause sterility in adult rats, mice, and hamsters via extensive degeneration of spermatogenic epithelium [8,9]. With regard to cadmium toxicity in mouse embryos, it can reduce the size of the genital ridge and the population of germ cells [10]. Cadmium was also found to induce both benign and malignant tumors at various sites in many species of experimental animals [11]. In living cells, protection against oxidative damage encompasses enzymatic and non-enzymatic antioxidant systems. The enzymatic system is represented in superoxide dismutase (SOD), catalase (CAT), glutathione peroxidase (GPx), glutathione reductase (GR), glutathione-S transferase (GSH), and esterases, whereas the non-enzymatic antioxidant system includes selenium, zinc, and reduced glutathione (GSH) [12]. Impairment of the enzymatic antioxidant system due to abnormal gene expression was mainly attributed to cadmium-induced oxidative stress [13,14]. Moreover, apoptotic cell death-induced by cadmium toxicity on rat testicle was reported results in decrease of its relative weight four days following cadmium injection [15].

Strawberry (*Fragaria ananassa*) is a widely-consumed fruit, belonging to the genus Fragaria with an attractive color, flavor and aroma. Strawberry extract contains a great variety of pigmented polyphenolic natural product compounds such as flavonoids, tannins, and some other compounds including ascorbic acid and essential oils with potent antioxidant effect [2,16]. It has been demonstrated that the crude extract of some berry fruits significantly reduced the oxidative stress in rat neuron cell line and brain tissue as well [17,18]. The ability of these extracts to cross the blood-brain barrier (BBB) has been confirmed [18]. These results prompted us to investigate in this study the protective effect of *Fragaria ananassa* crude extract on cadmium-induced oxidative stress in rat testes. Our results may pave the way toward the development of new therapeutic and preventive approaches against cadmium-induced oxidative damage of testicular function.

## 2. Results

**Figure 1:**
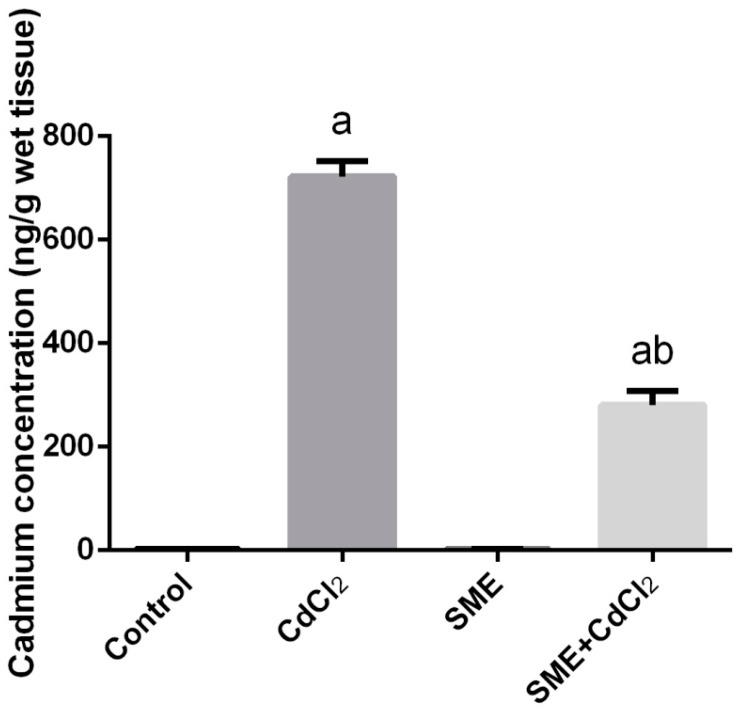
Effects of strawberry methanolic extract (SME) treatment on cadmium concentration in testis of rats exposed to cadmium (CdCl_2_) toxicity. All data are expressed as the mean ± SEM (*n* = 8). ^a^ Indicate significant change from the Control at *p* < 0.05; ^b^ indicate significant change from the CdCl_2_ at *p* < 0.05 using Duncan’s *post hoc* test.

CdCl_2_ injection in rats significantly (*p* < 0.05) elevated Cd concentration in testicular tissue compared to the control rats. The concentration of Cd in the testicular tissue was significantly (*p* < 0.05) mitigated by SME administration in CdCl_2_ exposed rats (Figure 1).

**Figure 2:**
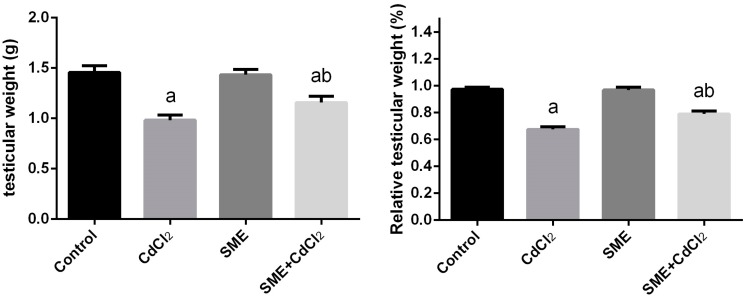
Effects of strawberry methanolic extract (SME) treatment on testis weight and relative testicular weight in rats exposed to cadmium (CdCl_2_) toxicity. All data are expressed as the mean ± SEM (*n* = 8). ^a^ Indicate significant change from the Control at *p* < 0.05; ^b^ indicate significant change from the CdCl_2_ at *p* < 0.05 using Duncan’s *post hoc* test.

Exposure to cadmium-induced toxicity for five days revealed a significant decrease in relative testicular weight of group 2 (received cadmium alone) by approx. 0.28 ± 0.02 (*p* < 0.05) when compared to control group. No marked reduction in the relative testicular weight of group 3 (received SME alone) was observed. However, group 4 (received cadmium and SME) indicated a slight decrease in this value (0.17 ± 0.02, *p* < 0.05) with that in the control group (Figure 2). 

**Figure 3:**
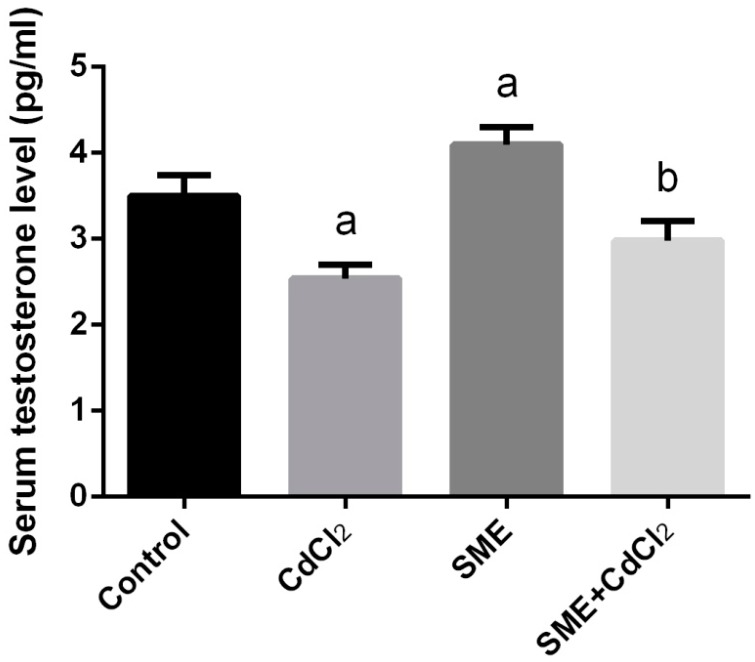
Effects of strawberry methanolic extract (SME) treatment on testosterone level in serum of rats exposed to cadmium (CdCl_2_) toxicity. All data are expressed as the mean ± SEM (*n* = 8). ^a^ Indicate significant change from the Control at *p* < 0.05; ^b^ indicate significant change from the CdCl_2_ at *p* < 0.05 using Duncan’s *post hoc* test.

Determining serum testosterone level showed a significant decrease of testosterone in group 2 (*p* < 0.05), whereas in group 3 the testosterone level was greatly increased compared to that of control (Figure 3). SME treated rats (group 4) revealed a slight decrease in the testosterone level.

**Figure 4:**
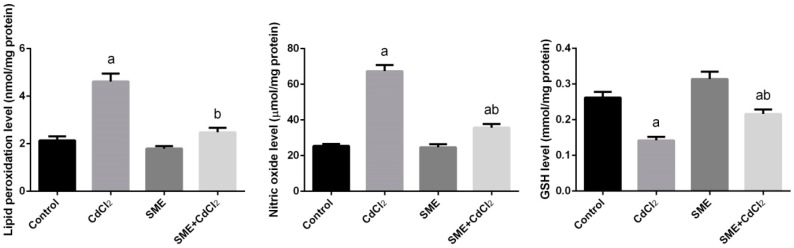
Effects of strawberry methanolic extract (SME) treatment on lipid peroxidation (LPO), nitric oxide (NO) levels and glutathione (GSH) content in testis of rats exposed to cadmium (CdCl_2_) toxicity. All data are expressed as the mean ± SEM (*n* = 8). ^a^ Indicate significant change from the Control at *p* < 0.05; ^b^ indicate significant change from the CdCl_2_ at *p* < 0.05 using Duncan’s *post hoc* test.

Intraperitoneal injection of cadmium alone resulted in significant increase of lipid peroxidation represented in the elevated level of both MDA and NO (Figure 4) in testicular homogenate when compared to control group. Conversely, oral administration of SME alone or in combination with cadmium exhibited a marked decrease in the level of MDA and NO, respectively. These values were found to be close to the normal values of control.

The level of GSH in testicular homogenate intoxicated with cadmium was significantly reduced incomparable to the control group (Figure 4). GSH level in group 3 provided only with SME was significantly increased compared to the level for the control rats. In group 4 treated with SME in response to cadmium intoxication, the level of GSH was significantly enhanced in the testicular tissue (*p* < 0.05).

**Figure 5:**
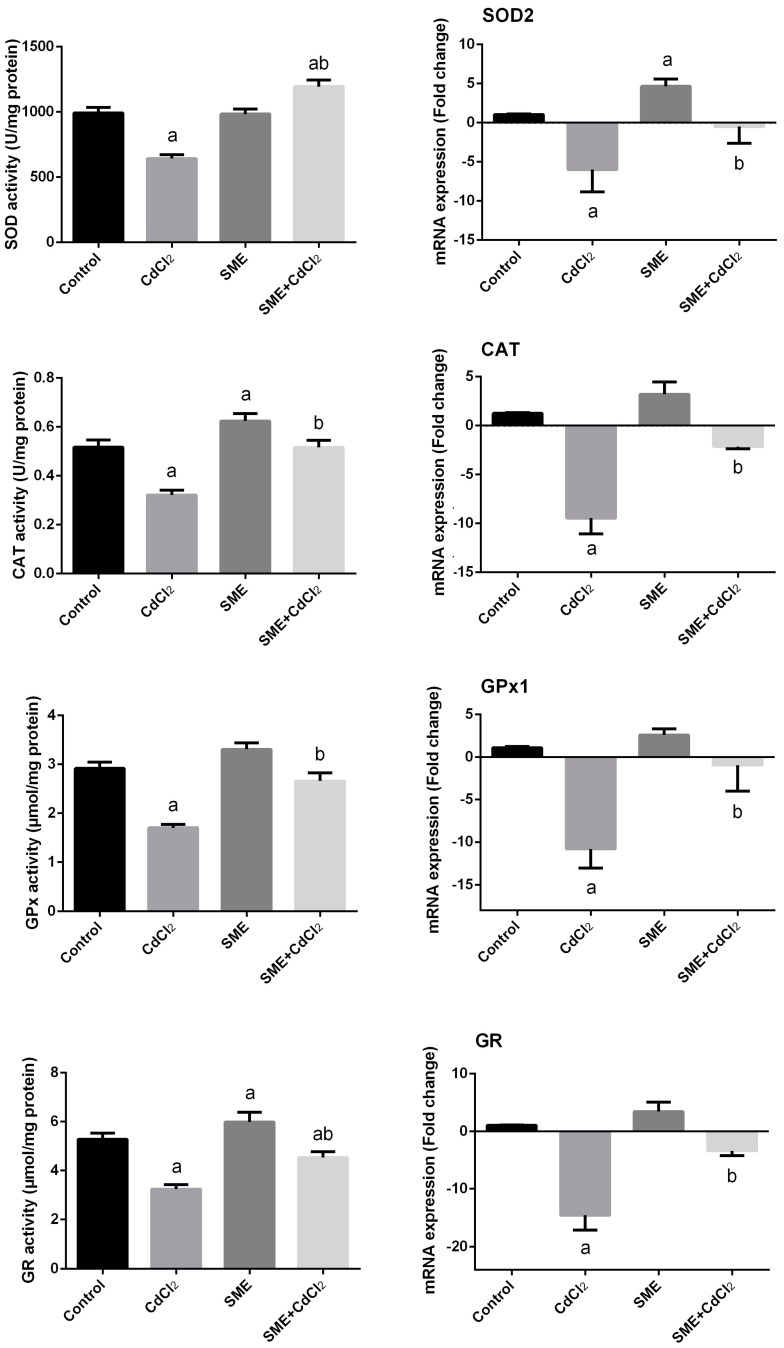
Effects of strawberry methanolic extract (SME) treatment on superoxide dismutase, catalase, glutathione peroxidase and glutathione reductase activities and their corresponding mRNA expressions in testis of rats exposed to cadmium (CdCl_2_) toxicity. Data of antioxidant enzyme activities are expressed as the mean ± SEM (*n* = 8) while the data of the mRNA expressions (mean ± SEM of triplicate assays) were normalized to the *GAPDH* mRNA level and are shown as the fold induction (in log2 scale) relative to the mRNA level in the controls. ^a^ Indicate significant change from the Control at *p* < 0.05; ^b^ indicate significant change from the CdCl_2_ at *p* < 0.05 using Duncan’s *post hoc* test.

The oxidative impact of cadmium on the testicular tissue was evaluated via determination of the activity of some antioxidant enzymes including SOD, CAT, GPx, and GR (Figure 5). In cadmium-exposed rats (group 2), the activity of all enzymes was significantly reduced in comparison to the control group (*p* < 0.05). CAT, GPx, and GR activity were greatly increased in group 3, which received SME alone, while the activity of SOD was recovered when compared to the control animals. The reduced GPx and GR activity due to cadmium intoxication in group 4 was significantly debilitated by SME treatment. Interestingly, the activity of CAT in group 4 returned to the normal value of the control and the SOD activity was markedly increased.

Gene expression analysis of these enzymes (*SOD2*, *CAT*, *GPX1*, and *GR* Figure 5) by RT-PCR revealed that all enzymes were downregulated in the testicular homogenate of group 2 as a result of i.p. injection of cadmium compared to the control animals. In the SME-group (group 3), all enzymes were markedly upregulated compared to that of the control. On the other hand, the SME-treated group exhibited a significant attenuation in the downregulation of the antioxidant enzymes compared to cadmium-induced oxidative damage in testes (group 4).

**Figure 6:**
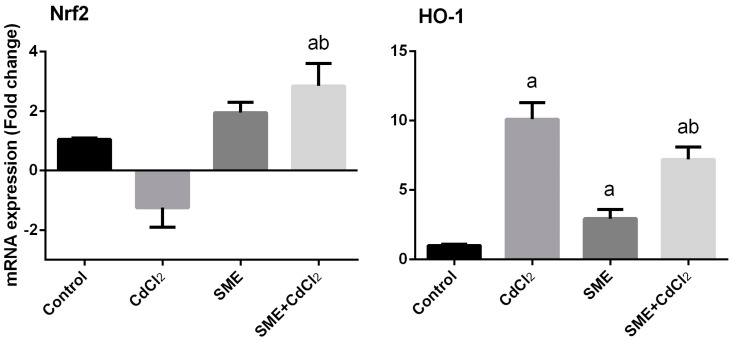
Effects of strawberry methanolic extract (SME) treatment on the mRNA expression of nuclear factor-erythroid 2-related factor 2 (*NFE2L2*) and heme oxygenase (*HMOX1*) in testis of rats exposed to cadmium (CdCl_2_) toxicity. Data of the mRNA expressions (mean ± SEM of triplicate assays) were normalized to the *GAPDH* mRNA level and are shown as the fold induction (in log2 scale) relative to the mRNA level in the controls. ^a^ Indicate significant change from the Control at *p* < 0.05; ^b^ indicate significant change from the CdCl_2_ at *p* < 0.05 using Duncan’s *post hoc* test.

To investigate the molecular mechanism by which SME upregulates the aforementioned antioxidant genes, the mRNA level of the stress sensor genes, *NFE2L2* and *HMOX1* was also quantified (Figure 6). In the cadmium-treated group (group 2) a significant downregulation of *NFE2L2* was detected, whereas incidentally upregulation in the level of *HMOX1* was observed when compared to the control group. Conversely, in group 3, treated with SME alone, the level of *NFE2L2* was obviously upregulated and the level of *HMOX1* was significantly increased. The group treated with SME in response to cadmium intoxication (group 4) possessed a significant attenuation of the oxidative damage-induced upon cadmium exposure via significant upregulation of *NFE2L2* and decreased expression level of *HMOX1* when compared to the Cd-intoxicated group. However, the *HMOX1* expression, when compared with the control rats, showed significant upregulation.

**Figure 7:**
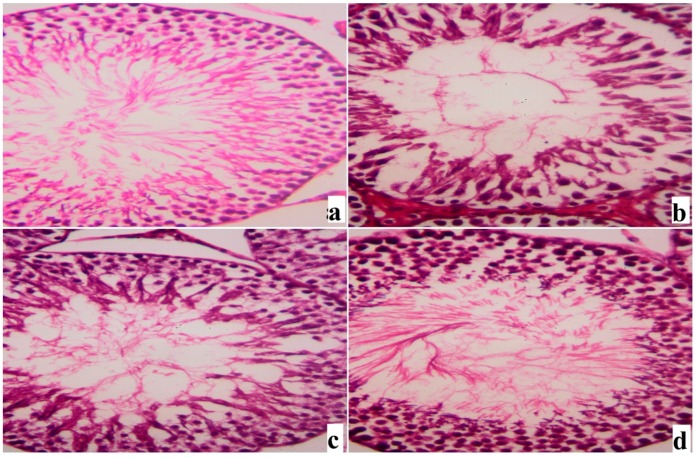
Testicular light micrographs of rats treated with SME and cadmium. (**a**) Photomicrograph of the testicular tissue of the control group showing normal seminiferous tubule with all stages of spermatogenic cells and the interstitial cells with Leydig cells filled the space between the seminiferous tubules; (**b**) Photomicrograph of the testicular tissue of rat from group intoxicated with cadmium showing degenerative changes in spermatogenic cells and detachment of the spermatogenic epithelium; (**c**) Photomicrograph of the testicular tissue of rat treated with SME alone showing normal histological structure; (**d**) Photomicrograph of the testicular tissue of rat treated with SME and cadmium showing partially preservation of spermatogenic epithelium in most seminiferous tubules. Sections were stained with hematoxylin and eosin (400×).

Histological examination of the testis of the control and SME-treated groups showed apparently normal testicular histology with normal and functional seminiferous tubules, all stages of the spermatogenic cells and the interstitial cells with Leydig cells filled the space between the seminiferous tubules (Figure 7a,c). By contrast, many histopathological alternations, a disorder of seminiferous tubule spermatogenic epithelium as well as a detachment of the spermatogenic epithelium from the basement membrane and a congestion of the interstitial spaces with degeneration of Leydig cells were observed in the rats treated with cadmium (Figure 7b). However, SME clearly restrained Cd-induced reproductive injury and the testis of rats in this group was preserved well (Figure 7d).

**Figure 8:**
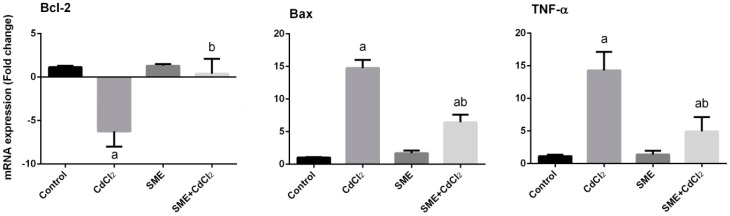
Effects of strawberry methanolic extract (SME) treatment on B-cell lymphoma 2 (*BCL2*), bcl-2-associated-X-protein (*BAX*), and tumor necrosis factor-α (*TNFA*) mRNA expressions in testis of rats exposed to cadmium (CdCl_2_) toxicity. Data of the mRNA expressions (mean ± SEM of triplicate assays) were normalized to the *GAPDH* mRNA level and are shown as the fold induction (in log2 scale) relative to the mRNA level in the controls. ^a^ Indicate significant change from the Control at *p* < 0.05; ^b^ indicate significant change from the CdCl_2_ at *p* < 0.05 using Duncan’s *post hoc* test.

The gene expression profile of *BCL2* was significantly upregulated in testis due to i.p. injection of cadmium (group 2), whereas its expression level didn’t change in group 3 that receive only SME compared to the control group. In group 4, which received SME in response to cadmium toxicity, the expression level of *BCL2* was greatly enhanced (Figure 8). Conversely, the expression level of Bax was obviously upregulated in the presence of cadmium (group 2) when compared to the control rats, however, SME markedly attenuates its expression level in the other groups (Figure 8). Analyzing the expression level of the *TNFA* gene revealed a significant upregulation as a result of cadmium intoxication in group 2. There were no significant changes in the expression level of the *TNFA* in group 3, where both were compared with the control group. The SME-treated rats (group 4) showed a significant attenuation in the expression level of *TNFA* (Figure 8). 

**Figure 9:**
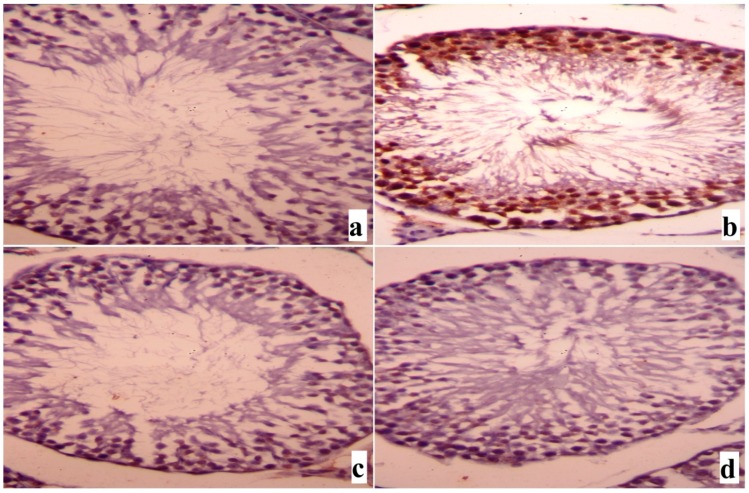
Testicular expression of TNF-α protein was detected using immunohistochemical staining in (**a**) control, (**b**) CdCl_2_-, (**c**) SME-, and (**d**) SME + CdCl_2_-treated rats. In the control and SME groups, brown staining cells, i.e., those stained with TNF-α, were sparse and weakly immunostained. By contrast, where the rats were treated with CdCl_2_, many testicular cells were inflamed and the brown-stained positive cells were increased markedly. In the SME + CdCl_2_ group, the number of inflamed testicular cells was markedly decreased (400×).

**Figure 10:**
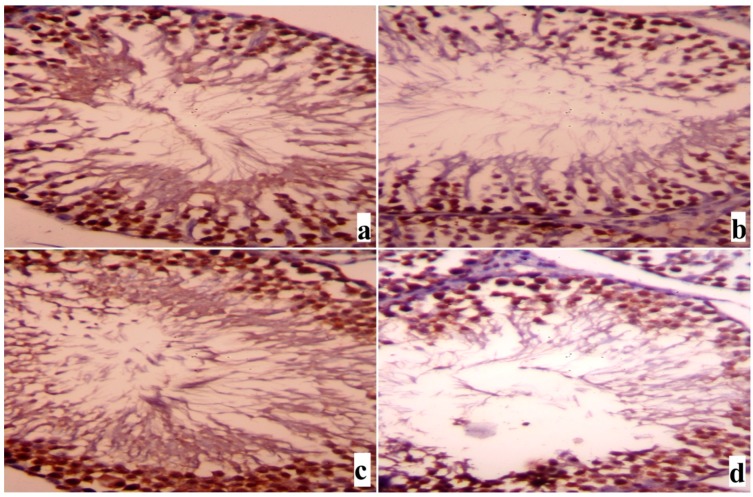
Testicular expression of PCNA was detected using immunohistochemical staining in (**a**) control, (**b**) CdCl_2_-, (**c**) SME-, and (**d**) SME + CdCl_2_-treated rats. In the control and SME groups, apoptotic testicular cells, i.e., those immunostained with PCNA, were sparse and weakly stained. By contrast, where the rats were treated with CdCl_2_, many testicular cells were apoptotic and the brown-stained positive cells were increased markedly. In the SME + CdCl_2_ group, the number of apoptotic testicular cells was decreased markedly (400×).

Immunohistochemistry investigation for the protective effect of SME on cadmium-induced alterations in the expression of TNF-α (Figure 9) and proliferating cell nuclear antigen (PCNA, Figure 10) in the testes of Wistar rats revealed that normal testicular tissue of both control and group 3 (Figure 9a,c for TNF-α and Figure 10a,c for PCNA). By contrast, the spermatogenic cells in group 2 were strongly affected by the deleterious effect of cadmium and possessed positive immunostaining for the increased level of TNF-α (Figure 9b), whereas few positive immunostained spermatogenic cells for PCNA (Figure 10b) were detected when compared to Figure 10a,c, respectively. In SME treated animals (group 4), the number of positively stained cells for TNF-α was markedly reduced (Figure 9d) and the number of immunoreactive spermatogenic cells for PCNA was significantly increased (Figure 10d).

## 3. Discussion

Cadmium is one of the most toxic heavy metals, and affects both animals and human via induction of cellular oxidative stress and subsequent elevation of free radicals. Strong evidence suggests the harmful effect of cadmium on testicular function, even with undetectable concentrations, making testes the most sensitive organ to cadmium toxicity [19,20]. In the present study, the protective effect of SME on the oxidative damage-induced by cadmium chloride in male Wistar rats’ testes was investigated. Several studies reported a dose-dependent decrease in the testicular weight as well as in the testicular function due to cadmium intoxication [21,22]. We showed a significant reduction in the relative testicular weight of the rats accompanied by a marked decrease of the serum testosterone level following injection with cadmium. Moreover, SME enhances serum testosterone level via reduction of cadmium concentration in the testicular homogenate. The reduced cadmium concentration of cadmium as a result of SME administration is suggested to be mainly assigned to the potential metal chelating property of the polyphenolic compounds, the main constituent of SME [23,24]. However, the exact mechanism by which the polyphenolic compounds of SME clear cadmium from the testes still remains enigmatic. It has been suggested that cadmium-induced oxidative stress via interaction with sulfhydryl (–SH) group of GSH results in an increased level of lipid peroxidation [25]. In the present study, systemic administration of cadmium revealed a pronounced increase in lipid peroxidation rate represented in the elevated level of MDA and NO in the testicular homogenate. This effect was accompanied by a significant reduction in GSH level. Conversely, SME treatment debilitated the formation of MDA and decreased the GSH level, possibly due to its antioxidant effect [26]. Cadmium was found to decrease the level of antioxidant enzymes as a result of oxidative damage and the subsequent generation of a considerable amount of ROS [27]. Consequently, we showed that cadmium was able to attenuate the level of all tested antioxidant enzymes including CAT, SOD, and GPx. The treatment group with SME alone (group 3) caused insignificant changes of the CAT, GPx, and GR activity when compared with the control group, whereas the activity of SOD was recovered to be comparable with the control. The combination of cadmium with SME possessed a great enhancement in the activity of CAT, GPx, and GR, while CAT activity was markedly increased compared to the control group. These results were further confirmed by the gene expression analysis of such enzymes. We suggest that the decrease in the activity of the antioxidant enzymes following administration of cadmium could be either due to transient enzyme inhibition by cadmium or due to cadmium-induced transcriptional inactivation of the corresponding genes [28]. Nrf2 is a transcription factor responsible for the transcriptional activation and regulation of the antioxidant genes via binding to their antioxidant response elements (ARE), thus preventing oxidative stress [29]. Nrf2 contributes to the expression of phase II enzymes those responsible for GSH synthesis, ROS quenching, and conjugation of pro-oxidative toxicants. HO-1 is an inducible stress-sensor enzyme expressed in response to a variety of oxidative challenges. It catalyzes the degradation of heme into biliverdin, which is subsequently converted to bilirubin by the action of bilirubin reductase [30]. Strong evidence suggests the induction of HO-1 by some non-heme inducers including endotoxins, metals, heat shock, inflammatory mediators, and prostaglandin [31]. This response was interpreted as a kind of cytoprotective mechanism against oxidative stress. The cytoprotective properties of HO-1 may arise due to the suppression of NADPH oxidase activity. It was shown that *NFE2L2* was significantly downregulated, while *HMOX1* was significantly upregulated in the testicular tissue as a result of cadmium intoxication. This obviously confirms the notorious oxidative damage of cadmium in testes. The increased expression level of *HMOX1* reflects its cytoprotective effect against cadmium-induced oxidative stress as an adaptation response. Moreover, the significant upregulation of *NFE2L2* level and the increased expression level of *HMOX1* in SME-treated groups explains the trigger mechanism by which SME upregulates the antioxidant enzymes and, while *HMOX1* expression is quite important for SME antioxidative activity, suppressing the *HMOX1* expression is not necessary for Cd-evoked oxidative stress in testicular tissue. TNF-α is a transmembrane protein/cytokine that is expressed on the surface of the activated macrophages in response to pathogen invasion. It is also considered to be an inflammatory mediator of both local and systemic inflammations [32]. TNF-α was suggested to play a central role in the production of interleukin-1 (IL-1) and some other mediators such as eicosanoids, NO, and ROS necessary for prolongation of the inflammatory response. In cadmium-treated group 2, the gene expression of both apoptotic *TNFA* and *BAX* genes was obviously upregulated, and the expression of the anti-apoptotic *BCL2* gene was significantly downregulated, confirming the apoptotic effect due to cadmium exposure. Treatment with SME alone debilitates all alterations induced by cadmium intoxication. However, in group 4 (SME & cadmium chloride) exhibited a significant reduction in apoptosis via decreasing the expression level of both *TNFA* and *BAX* and increasing the expression level of *BCL2*. Our results were in agreement with Al-Azemi et al., in which cadmium-induced oxidative damage of testicular tissues and stimulated the cells toward TNF apoptotic cell death [33]. Immunohistochemical investigation of rat testicular tissues indicated the destructive effect of cadmium chloride in group 2 characterized by significant upregulation of TNF-α. Treatment with SME alone (group3) didn’t change the expression level of TNF-α compared to the control. In group 4, which received SME and cadmium, the expression level of TNF-α was significantly reduced. PCNA is a nuclear protein that is involved in the replication and in the repair machinery of DNA. The expression of PCNA was found to be associated with the cell proliferation process [34]. Immunostaining detection of PCNA in testes is considered as a proliferative marker for the efficiency of spermatogenesis. Therefore, PCNA was used in this study to quantitatively analyze the spermatogenesis status. Administration of cadmium revealed few positive immunostained spermatogenic cells for PCNA when compared to the control group. These finding indicated that cadmium-induced oxidative damage of the testicular tissues is accompanied by depletion of the active DNA contents in the dividing spermatogenic cells. No significant changes were detected in the rat group that received SME alone. However, treatment with SME in response to cadmium intoxication results in a significant enhancement in the number of immune reactive spermatogenic cells for PCNA [35].

In the present work, we investigated the ability of *Fragaria ananassa* methanolic crude extract to ameliorate cadmium-induced oxidative damage in rat testicles. Oral administration of the crude extract to cadmium chloride treated group revealed a significant reduction of both LPO and NO levels, but marked increase of both testosterone and GSH levels was indicated. Moreover, gene expression of the antioxidant enzymes (*SOD2*, *CAT*, *GPX1*, and *GR*) was obviously enhanced as a result of NFE2L2 and *HMOX1* upregulation. Moreover, SME stimulated the inhibition of cadmium-induced apoptosis via downregulation of the apoptotic markers, *BAX* and *TNFA*. We suggest that the crude extract of *Fragaria ananassa* has an antioxidant protective effect against cadmium-induced oxidative stress. Our results pave the way toward the discovery and the development of lead structures against toxicity and infertility caused by environmental cadmium.

## 4. Materials and Methods

### 4.1. Sample Collection

The research was conducted during the period April–May, 2016, on freshly collected strawberry (*Fragaria ananassa*) samples from a market in Cairo, Egypt. Samples were taxonomically authenticated by the Botany Department, Faculty of Science, Helwan University, Cairo, Egypt by direct comparison with the already-available herbarium specimens.

### 4.2. Preparation of Strawberry Crude Extract

Methanol (70%) was added to 0.5 kg of fruit (1:10 *w*/*v*) followed by homogenization and continuous stirring for 48 h. For the preparation of crude extract, the homogenate was filtered and the extract was concentrated by vacuum rotary evaporator (IKA, Staufen im Breisgau, Germany). Strawberry methanolic extract (SME) was dissolved in water and kept at −20 °C. The average concentration of the polyphenolic and flavonoid compounds of SME was determined using standard methods to be 2110 and 1014 mg/100 g SME, respectively. Whereas, the total anthocyanin content was 380 mg/100 g SME.

### 4.3. Animals and Experimental Design

Thirty-two Adult male Wistar rats, 8–9 weeks old and weighing 160–180 g were purchased from the Animal facility of VACSERA Cairo, Egypt. The rats were housed at Zoology Department, Helwan University, Cairo, Egypt under standard laboratory conditions with a 12 h light/dark cycle and a fixed temperature (22 ± 25 °C), with access to pelleted rodent feed and water ad libitum. To investigate the protective effect of SME on cadmium-induced oxidative damage in rat testes, the study involved four randomly selected groups (*n* = 8). The first group was the control, while the other three were exposed to cadmium in different conditions. The first group (control) was injected with 0.9% saline solution (intraperitoneal, i.p.) for 5 days. The second group was subjected i.p. injection with 6.5 mg/kg CdCl_2_ dissolved in 0.9% physiological saline solution for 5 days. This dose has been previously reported to reveal no observable sign of toxicity [36]. The third group was supplemented with an oral administration of SME (250 mg/kg), whereas the fourth group was treated with SME one hour before i.p. injection with CdCl_2_ for 5 days. The supplemented dose of SME was calculated owing to our preliminary study, in which no sign of toxicity was clearly detected. Rats were sacrificed using ether then decapitated. Testes were isolated and homogenized in 10 mM phosphate puffer (pH 7.4). The homogenate was centrifuged for 10 min (10,000 rpm) at 4 °C and the supernatant was taken for further investigation. For the determination of serum testosterone, blood samples were also collected. The rules and guidelines governing handling and care of animals were approved by the Zoology department, Faculty of Science, Helwan University for the Laboratory Animal Care and in accordance with the National Institutes of Health (NIH) Guidelines for the Care and Use of Laboratory Animals 8th edition (NIH Publication No. 85-23 revised 1985).

### 4.4. Determination of Cadmium Concentration in Testes

Determination of cadmium concentration in testes was determined by atomic absorption spectrophotometer (Perkin Elmer 3100, PerkinElmer Inc, Shelton, CT, USA) as previously described [37]. Briefly, an appropriate weight of testes was digested with 3 mL of nitric acid (HNO_3_) in a tightly-capped 30-mL acid-washed polyethylene bottle. The bottle was left for 30 min at room temperature followed by incubation at 70 °C in a water bath for 3 h. Sample volume was completed into 5 mL with HNO_3_ and then treated with an equal volume of 10% *v*/*v* H_2_O_2_. Finally, the solutions were incubated at room temperature for 10 min. Afterward, an appropriate sample volume was injected into a graphite furnace at 228 nm. Samples were analyzed in duplicate and cadmium values were calculated from the standard curve on wet testes tissue basis in μg/g.

### 4.5. Testicular Weight Measurements

The testicular weight was determined using a sensitive weighing balance (Radwag, Model AS220/C/2, Clarkson laboratory and supply Inc., Chula Vista, CA, USA), whereas the relative testicular weight was calculated using the following formula:

$$Relative\;Testicular\;Weight\;\left(RTW\right)=\frac{Left\;Testis\;\left(LT\right)}{Body\;weight}\times 100$$

### 4.6. Biochemical Analysis

#### 4.6.1. Serum Testosterone

The amount of testosterone in the serum was assessed according to the instructions in the kit’s manual (Elecsys Testosterone Assay Kits, Roche Diagnostics, Mannheim, Germany), using a microplate reader (Chromate awareness 4300, Palm City, FL, USA). Samples were measured at 620 nm.

#### 4.6.2. Lipid Peroxidation (LPO)

LPO index in 10% testicular homogenate was performed by estimating the concentration of malondialdehyde (MDA) according to the method of Ohkawa et al. [38]. The developed color was measured as thiobarbituric acid reactive substances (TBARS) at 532 nm excitation and 555 nm emission. Accordingly, 100 mg of the testicular homogenate in phosphate buffer (pH 7.4) was mixed with 100 μL 100% trichloroacetic acid (TCA), 100 μL of sodium thioglycolate (1%), and 250 μL of 1 N HCl. The mixture was incubated at 100 °C for 20 min and then centrifuged for 10 min (4000 rpm). Spectrophotometric determination of TBA-MDA complex was determined in the supernatant.

#### 4.6.3. Nitric Oxide (NO) Level

Estimation of NO in testicular homogenate was carried out according to the method of Sastry et al. via quantification of its stable products represented in both nitrite (NO_2_) nitrate (NO_3_) levels [39]. Briefly, 100 μL of testicular homogenate was added to 400 μL carbonate buffer and a trace amount of activated copper-cadmium alloy. The mixture was incubated at room temperature with continuous shaking. To stop the reaction, the alloy was removed followed by addition of 100 μL of 0.35 M NaOH and 120 Mm ZnSO_4_. The mixture was subjected to a vigorous vortex and then allowed to stand for 10 min. The mixture was centrifuged for 10 min (4000 rpm) at room temperature. Griess reagent (50 μL) was added to 10 μL of the supernatant, incubated for 10 min, and finally, the absorbance was measured at 545 nm using a microplate ELISA reader.

#### 4.6.4. Reduced Glutathione (GSH)

Glutathione content in the tissue homogenate was determined using the method developed by Sedlak and Lindsay [40]. 250 μL of 10% testicular homogenate was added to 250 μL distilled water and 50 μL of 50% TCA. The mixture was subjected to successive shaking intervals for 15 min and then centrifuged at 3000 rpm for 10 min at room temperature. The supernatant (10 μL) was mixed with 400 μL 0.4 M Tris buffer (pH 8.9) and 10 μL of 5,5-dithio-*bis*-2-nitrobenzoic acid (DTNB) with continuous shaking. The developed color was measured at 512 nm by UV-VIS spectrophotometer (V-630; Jasco, Japan).

#### 4.6.5. Catalase (CAT) Activity

CAT activity was mainly assigned to the degradation rate of H_2_O_2_ per minute. The activity unit of CAT was expressed as U/mg protein [41]. The total volume (1 mL) of the enzymatic reaction mixture mainly consisted of 50 mM potassium phosphate (pH 7.0), 19 mM H_2_O_2_, and 50 μL of testicular homogenate supernatant. The molar extinction coefficient of H_2_O_2_ was monitored by UV-VIS spectrophotometer at 240 nm. One unit of enzyme activity was defined as the amount of H_2_O_2_ (μmol) consumed per min per milligram of tissue protein (U/mg protein).

#### 4.6.6. Superoxide Dismutase (SOD) Activity

Determination of SOD activity was measured using the method developed by Misra and Fridovich [42]. The method depends on the susceptibility of epinephrine toward oxidation at higher pH (10.2) and generation of adrenochrome and superoxide radicals (O_2_^−^). The SOD activity is then measured by the degree of inhibition of this reaction by decreasing the absorbance at 480 nm.

#### 4.6.7. Glutathione Peroxidase (GPx) Activity

GPx activity in testicular homogenate was assayed as previously described in [43]. The reaction volume was adjusted to 1.5 mL. Briefly, 200 μL of testicular supernatant was mixed with 1 mL of 75 mM phosphate buffer (pH 7.0), 10 mL of 150 mM glutathione, 10 mL of 340 U/mL glutathione reductase, 30 mL of 25 mM EDTA, 30 mL of 5 mM NADPH, 10 mL of 20% Triton X-100, and 50 μL of 7.5 mM H_2_O_2_. The oxidation of GSH is linked to the conversion of NADPH (extinction coefficient = 6.22 mM^−1^·cm^−1^) to NADP^+^ that monitored at 340 nm for 3 min. One unit of GPx activity was defined as the amount of GSH (nanomoles) oxidized per minute per milligram of protein (U/mg protein).

#### 4.6.8. Glutathione Reductase (GR) Activity

The activity of the GR enzyme was assayed by mixing the testicular supernatant (20 μL) with 0.44 mM oxidized glutathione (GSSG), 0.30 M EDTA, in 0.1 M phosphate buffer (pH 7.0). To start the enzymatic reaction, 0.036 M NADPH was added. The rate of NADPH oxidation was monitored by decreasing the absorbance at 340 nm as a function of time. One unit of enzyme was defined as the amount of enzyme required to oxidize 1 micromol of NADPH per minute [44].

### 4.7. Real Time-PCR

Extraction of total RNA from testes was performed with Trizol reagent and then converted to cDNA using the cDNA Synthesis Kit (Bio-Rad, Nazareth, Belgium) according to the manufacturer’s instructions. For gene expression analysis, cDNA of the oxidative stress enzyme markers (*CAT*, *SOD2*, *GPX1*, and *GR*), stress sensor proteins, nuclear factor-erythroid 2-related factor 2 (*NFE2L2*), and heme oxygenase-1 (*HMOX1*), as well as the anti-apoptotic B-cell lymphoma 2 (*BCL2*), pro-apoptotic bcl-2-associated X protein (*BAX*) markers, and the inflammatory marker, tumor necrosis factor α (*TNFA*), were used as a template for quantitative Real-Time PCR. QuantiFast SYBR Green RT-PCR kit (Qiagen, Hilden, Germany) and the corresponding forward and reverse primers indicated in Table 1 were implemented. Primers were obtained from (Jena Bioscience GmbH, Jena, Germany). All reactions were performed in triplicate using Applied Biosystems 7500 Instrument (Thermo Fisher Scientific Waltham, MA, USA). The PCR cycling conditions were set as follows: initial denaturation at 95 °C for 12 min, followed by 40 cycles of denaturation at 94 °C for 60 s and annealing at 55 °C for 60 s, extension at 72 °C for 90 s, and then held for a final extension at 72 °C for 10 min. The relative differences in gene expression between different groups were determined by delta-delta cycle threshold (Ct) method [45]. Glyceraldehyde-3-phosphate dehydrogenase (*GAPDH*) was used as a reference housekeeping gene.

### 4.8. Histological Procedures

The testes were excised from sacrificed animals and fixed in 10% formaldehyde/PBS for 24 h at room temperature. Testicular tissues were dehydrated using high grade alcohol, embedded in paraffin, and were sectioned at 5 μm thickness. Specimens were stained with hematoxylin and eosin stain. Finally, the slides were examined using a light microscope.

### 4.9. Immunohistochemistry Analysis

For immunohistochemistry analysis, the paraffinated sections were mounted on glass slides and deparaffinized. The antigen sites were unmasked by washing the slides with boiled water followed by treatment with 0.03% H_2_O_2_ in absolute methanol for 10 min to quench endogenous peroxidase activity. Section samples were incubated overnight at 4 °C with (1:50) polyclonal rabbit anti-TNF-α antibody and polyclonal rabbit anti-proliferating cell nuclear antigen (PCNA) antibody (Santa Cruz Biotechnology, Santa Cruz, CA, USA). To remove the unbound primary antibodies, section samples were washed with phosphate buffer saline (PBS). Afterwards, samples were incubated with goat-derived secondary anti-rabbit antibody conjugated to horseradish peroxidase at 37 °C for 30 min. Antigen-antibody interactions were finally detected by incubating samples with the chromogen 3,3′-diaminobenzidine tetrachloride (DAB-H_2_O_2_) at room temperature for 10 min as substrate. Testis sections were visualized using 400× magnification lens (Nikon Eclipse E200-LED, Tokyo, Japan). 

### 4.10. Statistical Analysis

Results were expressed as the mean ± standard error of the mean (SEM) of seven rats. Data comparison was analyzed by one-way analysis of variance (ANOVA). Post hoc Duncan multiple tests were done. *p* values < 0.05 were considered statistically significant.

## Figures and Tables

**Table 1:** Primer sequences of genes analyzed in real time PCR.

Name	Accession Number	Sense (5′–3′)	Antisense (5′–3′)
*GAPDH*	NM_017008.4	GCATCTTCTTGTGCAGTGCC	GATGGTGATGGGTTTCCCGT
*SOD2*	NM_001270850.1	AGCTGCACCACAGCAAGCAC	TCCACCACCCTTAGGGCTCA
*CAT*	NM_012520.2	TCCGGGATCTTTTTAACGCCATTG	TCGAGCACGGTAGGGACAGTTCAC
*GPX1*	NM_017006.2	CGGTTTCCCGTGCAATCAGT	ACACCGGGGACCAAATGATG
*GR*	NM_053906.2	TGCACTTCCCGGTAGGAAAC	GATCGCAACTGGGGTGAGAA
*NFE2L2*	NM_031789.2	GGTTGCCCACATTCCCAAAC	GGCTGGGAATATCCAGGGC
*HMOX1*	NM_012580.2	GCGAAACAAGCAGAACCCA	GCTCAGGATGAGTACCTCCCA
*BCL2*	NM_016993.1	CTGGTGGACAACATCGCTCTG	GGTCTGCTGACCTCACTTGTG
*BAX*	NM_017059.2	GGCGAATTGGCGATGAACTG	ATGGTTCTGATCAGCTCGGG
*TNFA*	XM_008772775.2	AGAACTCAGCGAGGACACCAA	GCTTGGTGGTTTGCTACGAC

The abbreviations of the genes; *GAPDH*: Glyceraldehyde 3-phosphate dehydrogenase; *SOD2*: Manganese-dependent superoxide dismutase (MnSOD); *CAT*: Catalase; *GPX1*: Glutathione peroxidase; *GR*: Glutathione reductase; *NFE2L2*: Nuclear factor-erythroid 2-related factor 2; *HMOX1*: Heme oxygenase-1; *BCL2*: B-cell lymphoma 2; *BAX*: bcl-2-associated X protein; *TNFA*: Tumor necrosis factor.
